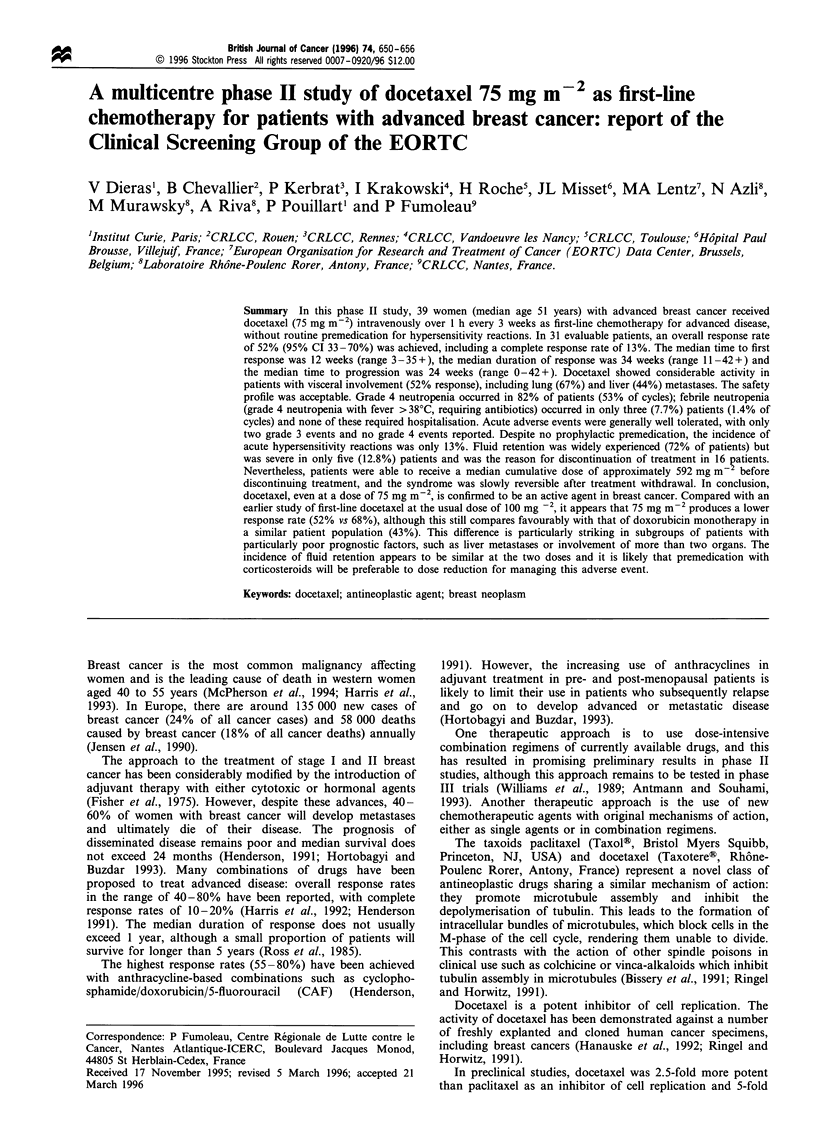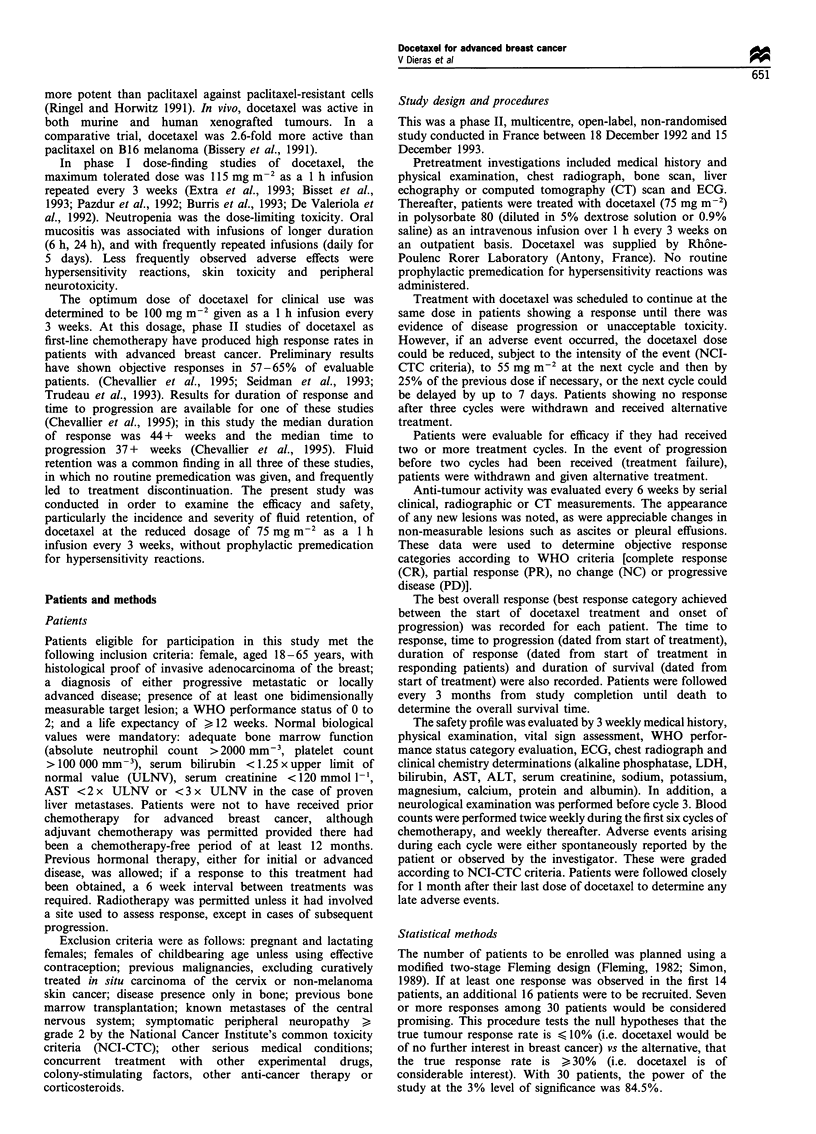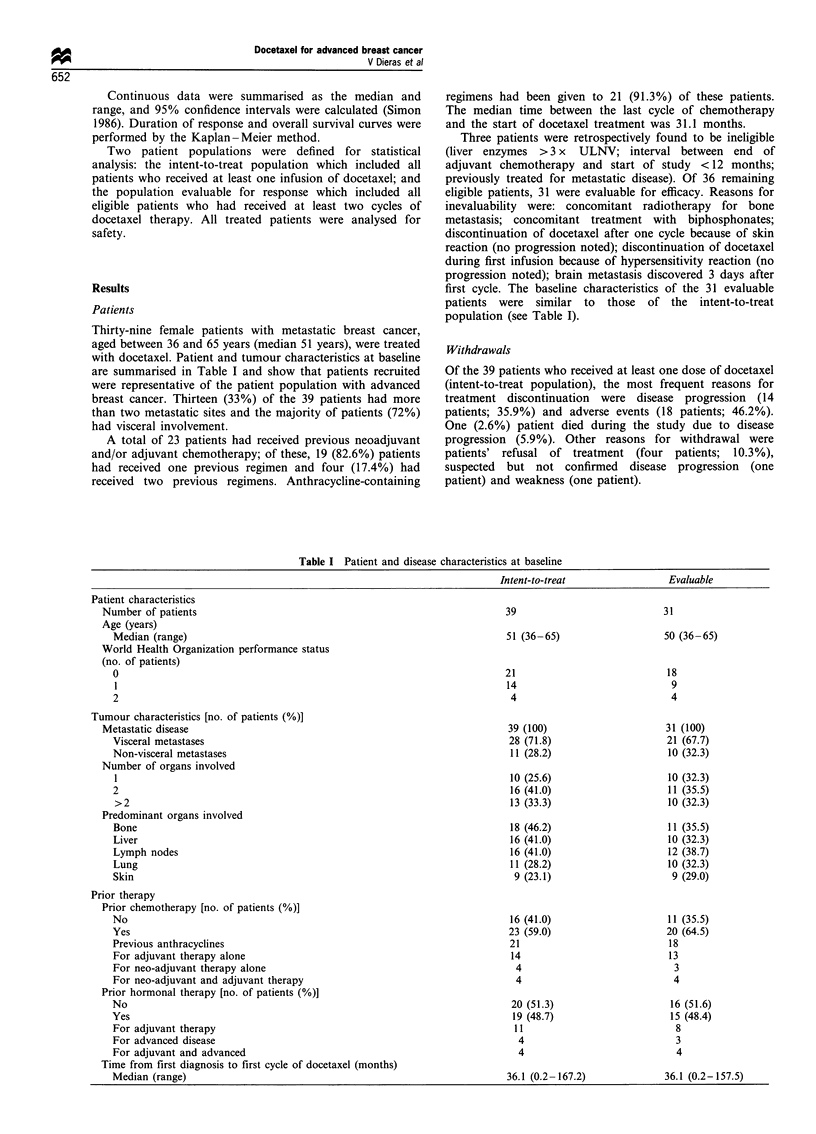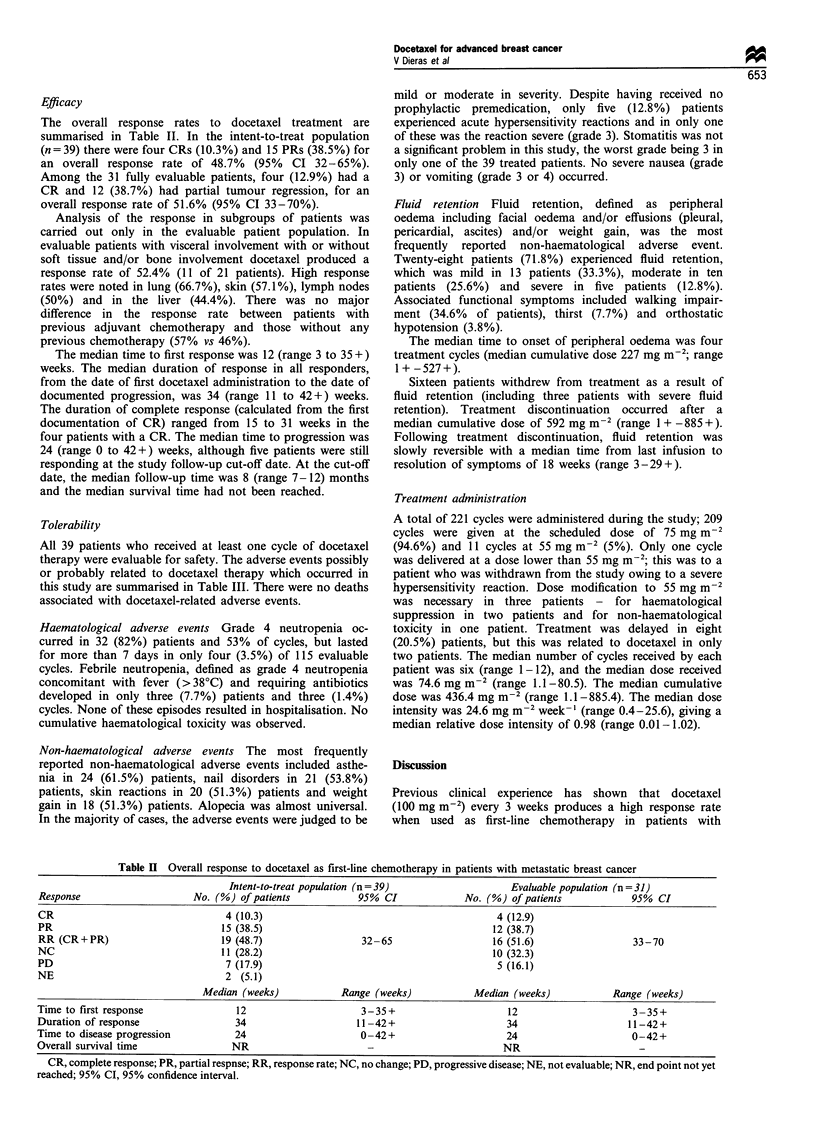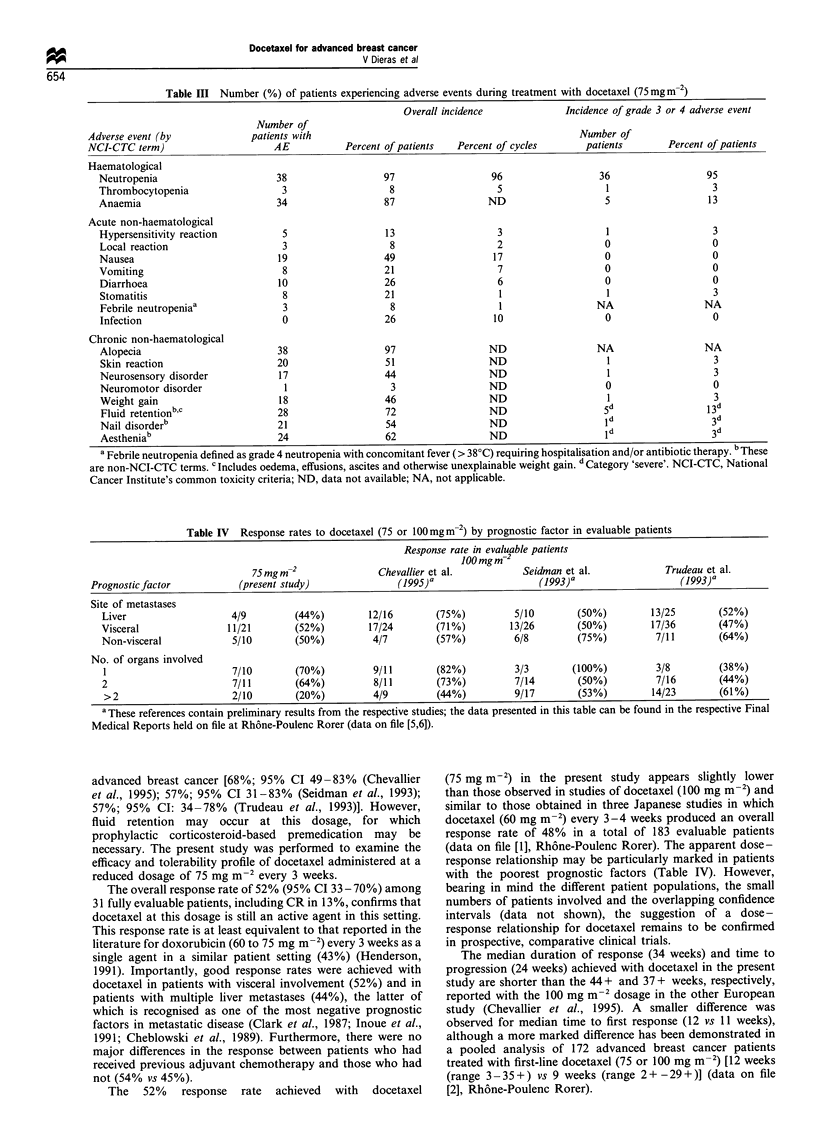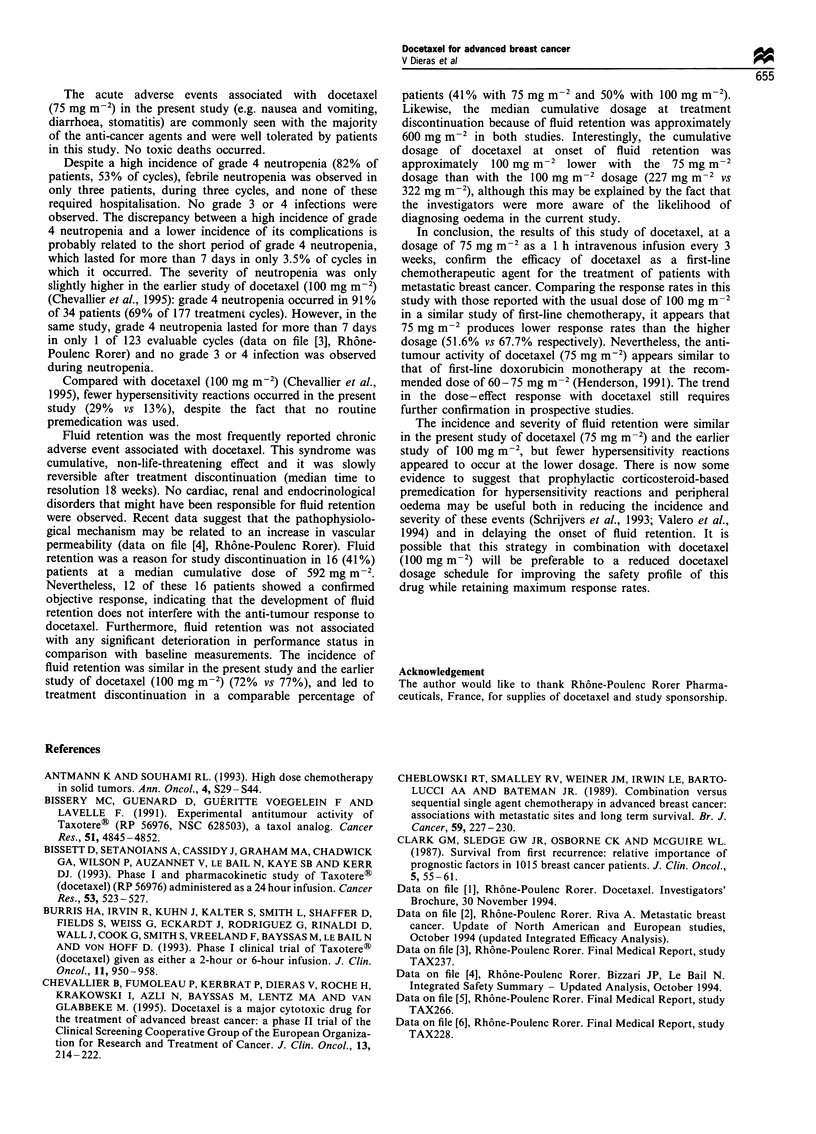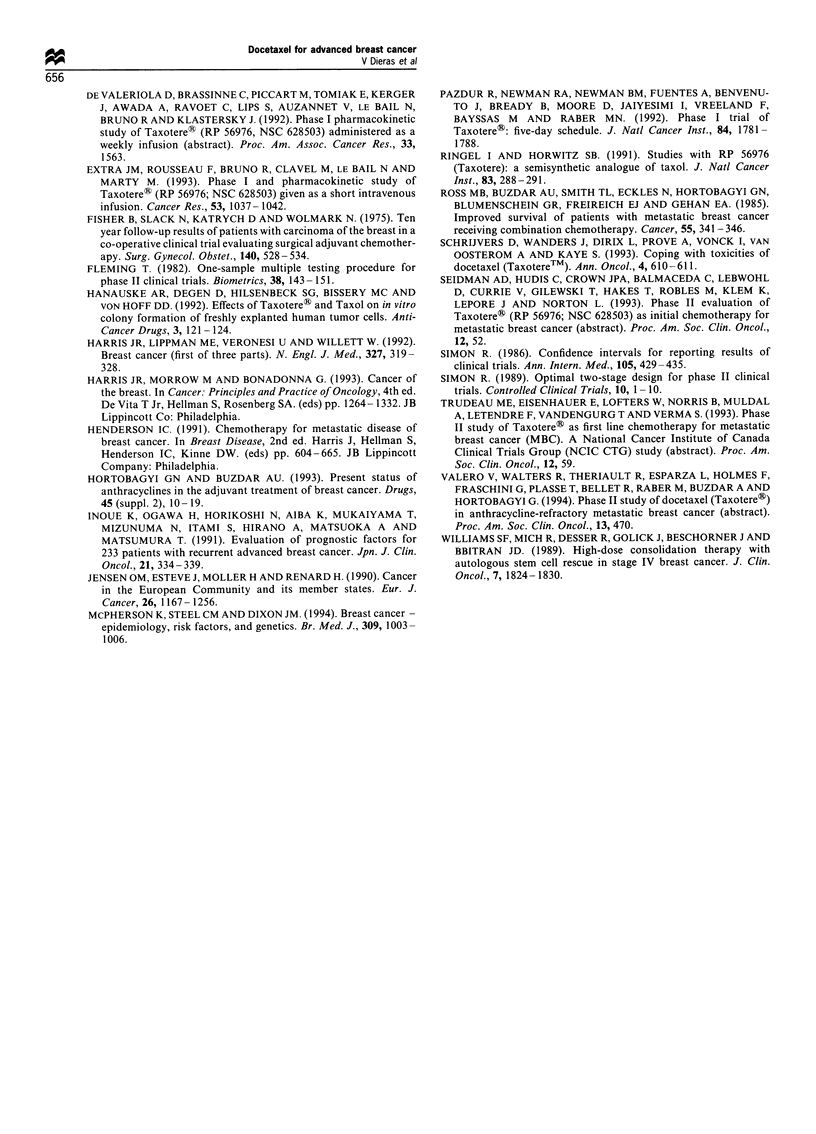# A multicentre phase II study of docetaxel 75 mg m-2 as first-line chemotherapy for patients with advanced breast cancer: report of the Clinical Screening Group of the EORTC. European Organization for Research and Treatment of Cancer.

**DOI:** 10.1038/bjc.1996.416

**Published:** 1996-08

**Authors:** V. Dieras, B. Chevallier, P. Kerbrat, I. Krakowski, H. Roche, J. L. Misset, M. A. Lentz, N. Azli, M. Murawsky, A. Riva, P. Pouillart, P. Fumoleau

**Affiliations:** Institut Curie, Paris, France.

## Abstract

In this phase II study, 39 women (median age 51 years) with advanced breast cancer received docetaxel (75 mg m-2) intravenously over 1 h every 3 weeks as first-line chemotherapy for advanced disease, without routine premedication for hypersensitivity reactions. In 31 evaluable patients, an overall response rate of 52% (95% CI 33-70%) was achieved, including a complete response rate of 13%. The median time to first response was 12 weeks (range 3-35+), the median duration of response was 34 weeks (range 11-42+) and the median time to progression was 24 weeks (range 0-42+). Docetaxel showed considerable activity in patients with visceral involvement (52% response), including lung (67%) and liver (44%) metastases. The safety profile was acceptable. Grade 4 neutropenia occurred in 82% of patients (53% of cycles); febrile neutropenia (grade 4 neutropenia with fever > 38 degrees C, requiring antibiotics) occurred in only three (7.7%) patients (1.4% of cycles) and none of these required hospitalisation. Acute adverse events were generally well tolerated, with only two grade 3 events and no grade 4 events reported. Despite no prophylactic premedication, the incidence of acute hypersensitivity reactions was only 13%. Fluid retention was widely experienced (72% of patients) but was severe in only five (12.8%) patients and was the reason for discontinuation of treatment in 16 patients. Nevertheless, patients were able to receive a median cumulative dose of approximately 592 mg m-2 before discontinuing treatment, and the syndrome was slowly reversible after treatment withdrawal. In conclusion, docetaxel, even at a dose of 75 mg m-2, is confirmed to be an active agent in breast cancer. Compared with an earlier study of first-line docetaxel at the usual dose of 100 mg-2, it appears that 75 mg m-2 produces a lower response rate (52% vs 68%), although this still compares favourably with that of doxorubicin monotherapy in a similar patient population (43%). This difference is particularly striking in subgroups of patients with particularly poor prognostic factors, such as liver metastases or involvement of more than two organs. The incidence of fluid retention appears to be similar at the two doses and, it is likely that premedication with corticosteroids will be preferable to dose reduction for managing this adverse event.


British Journal of Cancer (1996) 74, 650-656
© 1996 Stockton Press All rights reserved 0007-0920/96 $12.00

### A multicentre phase II study of docetaxel 75 mg m$^{-2}$ as first-line chemotherapy for patients with advanced breast cancer: report of the Clinical Screening Group of the EORTC

V Dieras[1], B Chevallier[2], P Kerbrat[3], I Krakowski[4], H Roche[5], JL Misset[6], MA Lentz[7], N Azli[8], M Murawsky[8], A Riva[8], P Pouillart[1] and P Fumoleau[9]

*[1]Institut Curie, Paris; [2]CRLCC, Rouen; [3]CRLCC, Rennes; [4]CRLCC, Vandoeuvre les Nancy; [5]CRLCC, Toulouse; [6]Hôpital Paul Brousse, Villejuif, France; [7]European Organisation for Research and Treatment of Cancer (EORTC) Data Center, Brussels, Belgium; [8]Laboratoire Rhône-Poulenc Rorer, Antony, France; [9]CRLCC, Nantes, France.*

**Summary** In this phase II study, 39 women (median age 51 years) with advanced breast cancer received docetaxel (75 mg m$^{-2}$) intravenously over 1 h every 3 weeks as first-line chemotherapy for advanced disease, without routine premedication for hypersensitivity reactions. In 31 evaluable patients, an overall response rate of 52% (95% CI 33–70%) was achieved, including a complete response rate of 13%. The median time to first response was 12 weeks (range 3–35+), the median duration of response was 34 weeks (range 11–42+) and the median time to progression was 24 weeks (range 0–42+). Docetaxel showed considerable activity in patients with visceral involvement (52% response), including lung (67%) and liver (44%) metastases. The safety profile was acceptable. Grade 4 neutropenia occurred in 82% of patients (53% of cycles); febrile neutropenia (grade 4 neutropenia with fever >38°C, requiring antibiotics) occurred in only three (7.7%) patients (1.4% of cycles) and none of these required hospitalisation. Acute adverse events were generally well tolerated, with only two grade 3 events and no grade 4 events reported. Despite no prophylactic premedication, the incidence of acute hypersensitivity reactions was only 13%. Fluid retention was widely experienced (72% of patients) but was severe in only five (12.8%) patients and was the reason for discontinuation of treatment in 16 patients. Nevertheless, patients were able to receive a median cumulative dose of approximately 592 mg m$^{-2}$ before discontinuing treatment, and the syndrome was slowly reversible after treatment withdrawal. In conclusion, docetaxel, even at a dose of 75 mg m$^{-2}$, is confirmed to be an active agent in breast cancer. Compared with an earlier study of first-line docetaxel at the usual dose of 100 mg $^{-2}$, it appears that 75 mg m$^{-2}$ produces a lower response rate (52% *vs* 68%), although this still compares favourably with that of doxorubicin monotherapy in a similar patient population (43%). This difference is particularly striking in subgroups of patients with particularly poor prognostic factors, such as liver metastases or involvement of more than two organs. The incidence of fluid retention appears to be similar at the two doses and it is likely that premedication with corticosteroids will be preferable to dose reduction for managing this adverse event.

**Keywords:** docetaxel; antineoplastic agent; breast neoplasm

Breast cancer is the most common malignancy affecting women and is the leading cause of death in western women aged 40 to 55 years (McPherson *et al.*, 1994; Harris *et al.*, 1993). In Europe, there are around 135 000 new cases of breast cancer (24% of all cancer cases) and 58 000 deaths caused by breast cancer (18% of all cancer deaths) annually (Jensen *et al.*, 1990).

The approach to the treatment of stage I and II breast cancer has been considerably modified by the introduction of adjuvant therapy with either cytotoxic or hormonal agents (Fisher *et al.*, 1975). However, despite these advances, 40–60% of women with breast cancer will develop metastases and ultimately die of their disease. The prognosis of disseminated disease remains poor and median survival does not exceed 24 months (Henderson, 1991; Hortobagyi and Buzdar 1993). Many combinations of drugs have been proposed to treat advanced disease: overall response rates in the range of 40–80% have been reported, with complete response rates of 10–20% (Harris *et al.*, 1992; Henderson 1991). The median duration of response does not usually exceed 1 year, although a small proportion of patients will survive for longer than 5 years (Ross *et al.*, 1985).

The highest response rates (55–80%) have been achieved with anthracycline-based combinations such as cyclophosphamide/doxorubicin/5-fluorouracil (CAF) (Henderson, 1991). However, the increasing use of anthracyclines in adjuvant treatment in pre- and post-menopausal patients is likely to limit their use in patients who subsequently relapse and go on to develop advanced or metastatic disease (Hortobagyi and Buzdar, 1993).

One therapeutic approach is to use dose-intensive combination regimens of currently available drugs, and this has resulted in promising preliminary results in phase II studies, although this approach remains to be tested in phase III trials (Williams *et al.*, 1989; Antmann and Souhami, 1993). Another therapeutic approach is the use of new chemotherapeutic agents with original mechanisms of action, either as single agents or in combination regimens.

The taxoids paclitaxel (Taxol®, Bristol Myers Squibb, Princeton, NJ, USA) and docetaxel (Taxotere®, Rhône-Poulenc Rorer, Antony, France) represent a novel class of antineoplastic drugs sharing a similar mechanism of action: they promote microtubule assembly and inhibit the depolymerisation of tubulin. This leads to the formation of intracellular bundles of microtubules, which block cells in the M-phase of the cell cycle, rendering them unable to divide. This contrasts with the action of other spindle poisons in clinical use such as colchicine or vinca-alkaloids which inhibit tubulin assembly in microtubules (Bissery *et al.*, 1991; Ringel and Horwitz, 1991).

Docetaxel is a potent inhibitor of cell replication. The activity of docetaxel has been demonstrated against a number of freshly explanted and cloned human cancer specimens, including breast cancers (Hanauske *et al.*, 1992; Ringel and Horwitz, 1991).

In preclinical studies, docetaxel was 2.5-fold more potent than paclitaxel as an inhibitor of cell replication and 5-fold

Correspondence: P Fumoleau, Centre Régionale de Lutte contre le Cancer, Nantes Atlantique-ICERC, Boulevard Jacques Monod, 44805 St Herblain-Cedex, France
Received 17 November 1995; revised 5 March 1996; accepted 21 March 1996

more potent than paclitaxel against paclitaxel-resistant cells (Ringel and Horwitz 1991). *In vivo*, docetaxel was active in both murine and human xenografted tumours. In a comparative trial, docetaxel was 2.6-fold more active than paclitaxel on B16 melanoma (Bissery *et al.*, 1991).

In phase I dose-finding studies of docetaxel, the maximum tolerated dose was 115 mg $m^{-2}$ as a 1 h infusion repeated every 3 weeks (Extra *et al.*, 1993; Bisset *et al.*, 1993; Pazdur *et al.*, 1992; Burris *et al.*, 1993; De Valeriola *et al.*, 1992). Neutropenia was the dose-limiting toxicity. Oral mucositis was associated with infusions of longer duration (6 h, 24 h), and with frequently repeated infusions (daily for 5 days). Less frequently observed adverse effects were hypersensitivity reactions, skin toxicity and peripheral neurotoxicity.

The optimum dose of docetaxel for clinical use was determined to be 100 mg $m^{-2}$ given as a 1 h infusion every 3 weeks. At this dosage, phase II studies of docetaxel as first-line chemotherapy have produced high response rates in patients with advanced breast cancer. Preliminary results have shown objective responses in 57–65% of evaluable patients. (Chevallier *et al.*, 1995; Seidman *et al.*, 1993; Trudeau *et al.*, 1993). Results for duration of response and time to progression are available for one of these studies (Chevallier *et al.*, 1995); in this study the median duration of response was 44+ weeks and the median time to progression 37+ weeks (Chevallier *et al.*, 1995). Fluid retention was a common finding in all three of these studies, in which no routine premedication was given, and frequently led to treatment discontinuation. The present study was conducted in order to examine the efficacy and safety, particularly the incidence and severity of fluid retention, of docetaxel at the reduced dosage of 75 mg $m^{-2}$ as a 1 h infusion every 3 weeks, without prophylactic premedication for hypersensitivity reactions.

### Patients and methods

### *Patients*

Patients eligible for participation in this study met the following inclusion criteria: female, aged 18–65 years, with histological proof of invasive adenocarcinoma of the breast; a diagnosis of either progressive metastatic or locally advanced disease; presence of at least one bidimensionally measurable target lesion; a WHO performance status of 0 to 2; and a life expectancy of $\geqslant$12 weeks. Normal biological values were mandatory: adequate bone marrow function (absolute neutrophil count >2000 $mm^{-3}$, platelet count >100 000 $mm^{-3}$), serum bilirubin <1.25×upper limit of normal value (ULNV), serum creatinine <120 mmol $l^{-1}$, AST <2× ULNV or <3× ULNV in the case of proven liver metastases. Patients were not to have received prior chemotherapy for advanced breast cancer, although adjuvant chemotherapy was permitted provided there had been a chemotherapy-free period of at least 12 months. Previous hormonal therapy, either for initial or advanced disease, was allowed; if a response to this treatment had been obtained, a 6 week interval between treatments was required. Radiotherapy was permitted unless it had involved a site used to assess response, except in cases of subsequent progression.

Exclusion criteria were as follows: pregnant and lactating females; females of childbearing age unless using effective contraception; previous malignancies, excluding curatively treated *in situ* carcinoma of the cervix or non-melanoma skin cancer; disease presence only in bone; previous bone marrow transplantation; known metastases of the central nervous system; symptomatic peripheral neuropathy $\geqslant$ grade 2 by the National Cancer Institute's common toxicity criteria (NCI-CTC); other serious medical conditions; concurrent treatment with other experimental drugs, colony-stimulating factors, other anti-cancer therapy or corticosteroids.

### *Study design and procedures*

This was a phase II, multicentre, open-label, non-randomised study conducted in France between 18 December 1992 and 15 December 1993.

Pretreatment investigations included medical history and physical examination, chest radiograph, bone scan, liver echography or computed tomography (CT) scan and ECG. Thereafter, patients were treated with docetaxel (75 mg $m^{-2}$) in polysorbate 80 (diluted in 5% dextrose solution or 0.9% saline) as an intravenous infusion over 1 h every 3 weeks on an outpatient basis. Docetaxel was supplied by Rhône-Poulenc Rorer Laboratory (Antony, France). No routine prophylactic premedication for hypersensitivity reactions was administered.

Treatment with docetaxel was scheduled to continue at the same dose in patients showing a response until there was evidence of disease progression or unacceptable toxicity. However, if an adverse event occurred, the docetaxel dose could be reduced, subject to the intensity of the event (NCI-CTC criteria), to 55 mg $m^{-2}$ at the next cycle and then by 25% of the previous dose if necessary, or the next cycle could be delayed by up to 7 days. Patients showing no response after three cycles were withdrawn and received alternative treatment.

Patients were evaluable for efficacy if they had received two or more treatment cycles. In the event of progression before two cycles had been received (treatment failure), patients were withdrawn and given alternative treatment.

Anti-tumour activity was evaluated every 6 weeks by serial clinical, radiographic or CT measurements. The appearance of any new lesions was noted, as were appreciable changes in non-measurable lesions such as ascites or pleural effusions. These data were used to determine objective response categories according to WHO criteria [complete response (CR), partial response (PR), no change (NC) or progressive disease (PD)].

The best overall response (best response category achieved between the start of docetaxel treatment and onset of progression) was recorded for each patient. The time to response, time to progression (dated from start of treatment), duration of response (dated from start of treatment in responding patients) and duration of survival (dated from start of treatment) were also recorded. Patients were followed every 3 months from study completion until death to determine the overall survival time.

The safety profile was evaluated by 3 weekly medical history, physical examination, vital sign assessment, WHO performance status category evaluation, ECG, chest radiograph and clinical chemistry determinations (alkaline phosphatase, LDH, bilirubin, AST, ALT, serum creatinine, sodium, potassium, magnesium, calcium, protein and albumin). In addition, a neurological examination was performed before cycle 3. Blood counts were performed twice weekly during the first six cycles of chemotherapy, and weekly thereafter. Adverse events arising during each cycle were either spontaneously reported by the patient or observed by the investigator. These were graded according to NCI-CTC criteria. Patients were followed closely for 1 month after their last dose of docetaxel to determine any late adverse events.

### *Statistical methods*

The number of patients to be enrolled was planned using a modified two-stage Fleming design (Fleming, 1982; Simon, 1989). If at least one response was observed in the first 14 patients, an additional 16 patients were to be recruited. Seven or more responses among 30 patients would be considered promising. This procedure tests the null hypotheses that the true tumour response rate is $\leqslant$10% (i.e. docetaxel would be of no further interest in breast cancer) *vs* the alternative, that the true response rate is $\geqslant$30% (i.e. docetaxel is of considerable interest). With 30 patients, the power of the study at the 3% level of significance was 84.5%.

Continuous data were summarised as the median and range, and 95% confidence intervals were calculated (Simon 1986). Duration of response and overall survival curves were performed by the Kaplan–Meier method.

Two patient populations were defined for statistical analysis: the intent-to-treat population which included all patients who received at least one infusion of docetaxel; and the population evaluable for response which included all eligible patients who had received at least two cycles of docetaxel therapy. All treated patients were analysed for safety.

### Results

### *Patients*

Thirty-nine female patients with metastatic breast cancer, aged between 36 and 65 years (median 51 years), were treated with docetaxel. Patient and tumour characteristics at baseline are summarised in Table I and show that patients recruited were representative of the patient population with advanced breast cancer. Thirteen (33%) of the 39 patients had more than two metastatic sites and the majority of patients (72%) had visceral involvement.

A total of 23 patients had received previous neoadjuvant and/or adjuvant chemotherapy; of these, 19 (82.6%) patients had received one previous regimen and four (17.4%) had received two previous regimens. Anthracycline-containing regimens had been given to 21 (91.3%) of these patients. The median time between the last cycle of chemotherapy and the start of docetaxel treatment was 31.1 months.

Three patients were retrospectively found to be ineligible (liver enzymes >3× ULNV; interval between end of adjuvant chemotherapy and start of study <12 months; previously treated for metastatic disease). Of 36 remaining eligible patients, 31 were evaluable for efficacy. Reasons for inevaluability were: concomitant radiotherapy for bone metastasis; concomitant treatment with biphosphonates; discontinuation of docetaxel after one cycle because of skin reaction (no progression noted); discontinuation of docetaxel during first infusion because of hypersensitivity reaction (no progression noted); brain metastasis discovered 3 days after first cycle. The baseline characteristics of the 31 evaluable patients were similar to those of the intent-to-treat population (see Table I).

### *Withdrawals*

Of the 39 patients who received at least one dose of docetaxel (intent-to-treat population), the most frequent reasons for treatment discontinuation were disease progression (14 patients; 35.9%) and adverse events (18 patients; 46.2%). One (2.6%) patient died during the study due to disease progression (5.9%). Other reasons for withdrawal were patients' refusal of treatment (four patients; 10.3%), suspected but not confirmed disease progression (one patient) and weakness (one patient).

**Table I** Patient and disease characteristics at baseline

| | *Intent-to-treat* | *Evaluable* |
|---|---|---|
| Patient characteristics | | |
| Number of patients | 39 | 31 |
| Age (years) | | |
| Median (range) | 51 (36–65) | 50 (36–65) |
| World Health Organization performance status (no. of patients) | | |
| 0 | 21 | 18 |
| 1 | 14 | 9 |
| 2 | 4 | 4 |
| Tumour characteristics [no. of patients (%)] | | |
| Metastatic disease | 39 (100) | 31 (100) |
| Visceral metastases | 28 (71.8) | 21 (67.7) |
| Non-visceral metastases | 11 (28.2) | 10 (32.3) |
| Number of organs involved | | |
| 1 | 10 (25.6) | 10 (32.3) |
| 2 | 16 (41.0) | 11 (35.5) |
| >2 | 13 (33.3) | 10 (32.3) |
| Predominant organs involved | | |
| Bone | 18 (46.2) | 11 (35.5) |
| Liver | 16 (41.0) | 10 (32.3) |
| Lymph nodes | 16 (41.0) | 12 (38.7) |
| Lung | 11 (28.2) | 10 (32.3) |
| Skin | 9 (23.1) | 9 (29.0) |
| Prior therapy | | |
| Prior chemotherapy [no. of patients (%)] | | |
| No | 16 (41.0) | 11 (35.5) |
| Yes | 23 (59.0) | 20 (64.5) |
| Previous anthracyclines | 21 | 18 |
| For adjuvant therapy alone | 14 | 13 |
| For neo-adjuvant therapy alone | 4 | 3 |
| For neo-adjuvant and adjuvant therapy | 4 | 4 |
| Prior hormonal therapy [no. of patients (%)] | | |
| No | 20 (51.3) | 16 (51.6) |
| Yes | 19 (48.7) | 15 (48.4) |
| For adjuvant therapy | 11 | 8 |
| For advanced disease | 4 | 3 |
| For adjuvant and advanced | 4 | 4 |
| Time from first diagnosis to first cycle of docetaxel (months) | | |
| Median (range) | 36.1 (0.2–167.2) | 36.1 (0.2–157.5) |

### *Efficacy*

The overall response rates to docetaxel treatment are summarised in Table II. In the intent-to-treat population ($n=39$) there were four CRs (10.3%) and 15 PRs (38.5%) for an overall response rate of 48.7% (95% CI 32–65%). Among the 31 fully evaluable patients, four (12.9%) had a CR and 12 (38.7%) had partial tumour regression, for an overall response rate of 51.6% (95% CI 33–70%).

Analysis of the response in subgroups of patients was carried out only in the evaluable patient population. In evaluable patients with visceral involvement with or without soft tissue and/or bone involvement docetaxel produced a response rate of 52.4% (11 of 21 patients). High response rates were noted in lung (66.7%), skin (57.1%), lymph nodes (50%) and in the liver (44.4%). There was no major difference in the response rate between patients with previous adjuvant chemotherapy and those without any previous chemotherapy (57% *vs* 46%).

The median time to first response was 12 (range 3 to 35+) weeks. The median duration of response in all responders, from the date of first docetaxel administration to the date of documented progression, was 34 (range 11 to 42+) weeks. The duration of complete response (calculated from the first documentation of CR) ranged from 15 to 31 weeks in the four patients with a CR. The median time to progression was 24 (range 0 to 42+) weeks, although five patients were still responding at the study follow-up cut-off date. At the cut-off date, the median follow-up time was 8 (range 7–12) months and the median survival time had not been reached.

### *Tolerability*

All 39 patients who received at least one cycle of docetaxel therapy were evaluable for safety. The adverse events possibly or probably related to docetaxel therapy which occurred in this study are summarised in Table III. There were no deaths associated with docetaxel-related adverse events.

*Haematological adverse events* Grade 4 neutropenia occurred in 32 (82%) patients and 53% of cycles, but lasted for more than 7 days in only four (3.5%) of 115 evaluable cycles. Febrile neutropenia, defined as grade 4 neutropenia concomitant with fever (>38°C) and requiring antibiotics developed in only three (7.7%) patients and three (1.4%) cycles. None of these episodes resulted in hospitalisation. No cumulative haematological toxicity was observed.

*Non-haematological adverse events* The most frequently reported non-haematological adverse events included asthenia in 24 (61.5%) patients, nail disorders in 21 (53.8%) patients, skin reactions in 20 (51.3%) patients and weight gain in 18 (51.3%) patients. Alopecia was almost universal. In the majority of cases, the adverse events were judged to be mild or moderate in severity. Despite having received no prophylactic premedication, only five (12.8%) patients experienced acute hypersensitivity reactions and in only one of these was the reaction severe (grade 3). Stomatitis was not a significant problem in this study, the worst grade being 3 in only one of the 39 treated patients. No severe nausea (grade 3) or vomiting (grade 3 or 4) occurred.

*Fluid retention* Fluid retention, defined as peripheral oedema including facial oedema and/or effusions (pleural, pericardial, ascites) and/or weight gain, was the most frequently reported non-haematological adverse event. Twenty-eight patients (71.8%) experienced fluid retention, which was mild in 13 patients (33.3%), moderate in ten patients (25.6%) and severe in five patients (12.8%). Associated functional symptoms included walking impairment (34.6% of patients), thirst (7.7%) and orthostatic hypotension (3.8%).

The median time to onset of peripheral oedema was four treatment cycles (median cumulative dose 227 mg $m^{-2}$; range 1+–527+).

Sixteen patients withdrew from treatment as a result of fluid retention (including three patients with severe fluid retention). Treatment discontinuation occurred after a median cumulative dose of 592 mg $m^{-2}$ (range 1+–885+). Following treatment discontinuation, fluid retention was slowly reversible with a median time from last infusion to resolution of symptoms of 18 weeks (range 3–29+).

### *Treatment administration*

A total of 221 cycles were administered during the study; 209 cycles were given at the scheduled dose of 75 mg $m^{-2}$ (94.6%) and 11 cycles at 55 mg $m^{-2}$ (5%). Only one cycle was delivered at a dose lower than 55 mg $m^{-2}$; this was to a patient who was withdrawn from the study owing to a severe hypersensitivity reaction. Dose modification to 55 mg $m^{-2}$ was necessary in three patients – for haematological suppression in two patients and for non-haematological toxicity in one patient. Treatment was delayed in eight (20.5%) patients, but this was related to docetaxel in only two patients. The median number of cycles received by each patient was six (range 1–12), and the median dose received was 74.6 mg $m^{-2}$ (range 1.1–80.5). The median cumulative dose was 436.4 mg $m^{-2}$ (range 1.1–885.4). The median dose intensity was 24.6 mg $m^{-2}$ $week^{-1}$ (range 0.4–25.6), giving a median relative dose intensity of 0.98 (range 0.01–1.02).

### Discussion

Previous clinical experience has shown that docetaxel (100 mg $m^{-2}$) every 3 weeks produces a high response rate when used as first-line chemotherapy in patients with

**Table II** Overall response to docetaxel as first-line chemotherapy in patients with metastatic breast cancer

| Response | Intent-to-treat population (n=39) No. (%) of patients | 95% CI | Evaluable population (n=31) No. (%) of patients | 95% CI |
|---|---|---|---|---|
| CR | 4 (10.3) | | 4 (12.9) | |
| PR | 15 (38.5) | | 12 (38.7) | |
| RR (CR+PR) | 19 (48.7) | 32–65 | 16 (51.6) | 33–70 |
| NC | 11 (28.2) | | 10 (32.3) | |
| PD | 7 (17.9) | | 5 (16.1) | |
| NE | 2 (5.1) | | | |
| | *Median (weeks)* | *Range (weeks)* | *Median (weeks)* | *Range (weeks)* |
| Time to first response | 12 | 3–35+ | 12 | 3–35+ |
| Duration of response | 34 | 11–42+ | 34 | 11–42+ |
| Time to disease progression | 24 | 0–42+ | 24 | 0–42+ |
| Overall survival time | NR | – | NR | – |

CR, complete response; PR, partial respnse; RR, response rate; NC, no change; PD, progressive disease; NE, not evaluable; NR, end point not yet reached; 95% CI, 95% confidence interval.

**Table III** Number (%) of patients experiencing adverse events during treatment with docetaxel (75 mg m$^{-2}$)

| Adverse event (by NCI-CTC term) | Number of patients with AE | Overall incidence: Percent of patients | Overall incidence: Percent of cycles | Incidence of grade 3 or 4 adverse event: Number of patients | Incidence of grade 3 or 4 adverse event: Percent of patients |
|---|---|---|---|---|---|
| Haematological | | | | | |
| Neutropenia | 38 | 97 | 96 | 36 | 95 |
| Thrombocytopenia | 3 | 8 | 5 | 1 | 3 |
| Anaemia | 34 | 87 | ND | 5 | 13 |
| Acute non-haematological | | | | | |
| Hypersensitivity reaction | 5 | 13 | 3 | 1 | 3 |
| Local reaction | 3 | 8 | 2 | 0 | 0 |
| Nausea | 19 | 49 | 17 | 0 | 0 |
| Vomiting | 8 | 21 | 7 | 0 | 0 |
| Diarrhoea | 10 | 26 | 6 | 0 | 0 |
| Stomatitis | 8 | 21 | 1 | 1 | 3 |
| Febrile neutropenia[a] | 3 | 8 | 1 | NA | NA |
| Infection | 0 | 26 | 10 | 0 | 0 |
| Chronic non-haematological | | | | | |
| Alopecia | 38 | 97 | ND | NA | NA |
| Skin reaction | 20 | 51 | ND | 1 | 3 |
| Neurosensory disorder | 17 | 44 | ND | 1 | 3 |
| Neuromotor disorder | 1 | 3 | ND | 0 | 0 |
| Weight gain | 18 | 46 | ND | 1 | 3 |
| Fluid retention[b,c] | 28 | 72 | ND | 5[d] | 13[d] |
| Nail disorder[b] | 21 | 54 | ND | 1[d] | 3[d] |
| Aesthenia[b] | 24 | 62 | ND | 1[d] | 3[d] |

[a] Febrile neutropenia defined as grade 4 neutropenia with concomitant fever (>38°C) requiring hospitalisation and/or antibiotic therapy. [b] These are non-NCI-CTC terms. [c] Includes oedema, effusions, ascites and otherwise unexplainable weight gain. [d] Category 'severe'. NCI-CTC, National Cancer Institute's common toxicity criteria; ND, data not available; NA, not applicable.

**Table IV** Response rates to docetaxel (75 or 100 mg m$^{-2}$) by prognostic factor in evaluable patients

| Prognostic factor | 75 mg m$^{-2}$ (present study) | | 100 mg m$^{-2}$: Chevallier et al. (1995)[a] | | 100 mg m$^{-2}$: Seidman et al. (1993)[a] | | 100 mg m$^{-2}$: Trudeau et al. (1993)[a] | |
|---|---|---|---|---|---|---|---|---|
| Site of metastases | | | | | | | | |
| Liver | 4/9 | (44%) | 12/16 | (75%) | 5/10 | (50%) | 13/25 | (52%) |
| Visceral | 11/21 | (52%) | 17/24 | (71%) | 13/26 | (50%) | 17/36 | (47%) |
| Non-visceral | 5/10 | (50%) | 4/7 | (57%) | 6/8 | (75%) | 7/11 | (64%) |
| No. of organs involved | | | | | | | | |
| 1 | 7/10 | (70%) | 9/11 | (82%) | 3/3 | (100%) | 3/8 | (38%) |
| 2 | 7/11 | (64%) | 8/11 | (73%) | 7/14 | (50%) | 7/16 | (44%) |
| >2 | 2/10 | (20%) | 4/9 | (44%) | 9/17 | (53%) | 14/23 | (61%) |

Response rate in evaluable patients.

[a] These references contain preliminary results from the respective studies; the data presented in this table can be found in the respective Final Medical Reports held on file at Rhône-Poulenc Rorer (data on file [5,6]).

advanced breast cancer [68%; 95% CI 49–83% (Chevallier *et al.*, 1995); 57%; 95% CI 31–83% (Seidman *et al.*, 1993); 57%; 95% CI: 34–78% (Trudeau *et al.*, 1993)]. However, fluid retention may occur at this dosage, for which prophylactic corticosteroid-based premedication may be necessary. The present study was performed to examine the efficacy and tolerability profile of docetaxel administered at a reduced dosage of 75 mg m$^{-2}$ every 3 weeks.

The overall response rate of 52% (95% CI 33–70%) among 31 fully evaluable patients, including CR in 13%, confirms that docetaxel at this dosage is still an active agent in this setting. This response rate is at least equivalent to that reported in the literature for doxorubicin (60 to 75 mg m$^{-2}$) every 3 weeks as a single agent in a similar patient setting (43%) (Henderson, 1991). Importantly, good response rates were achieved with docetaxel in patients with visceral involvement (52%) and in patients with multiple liver metastases (44%), the latter of which is recognised as one of the most negative prognostic factors in metastatic disease (Clark *et al.*, 1987; Inoue *et al.*, 1991; Cheblowski *et al.*, 1989). Furthermore, there were no major differences in the response between patients who had received previous adjuvant chemotherapy and those who had not (54% *vs* 45%).

The 52% response rate achieved with docetaxel (75 mg m$^{-2}$) in the present study appears slightly lower than those observed in studies of docetaxel (100 mg m$^{-2}$) and similar to those obtained in three Japanese studies in which docetaxel (60 mg m$^{-2}$) every 3–4 weeks produced an overall response rate of 48% in a total of 183 evaluable patients (data on file [1], Rhône-Poulenc Rorer). The apparent dose–response relationship may be particularly marked in patients with the poorest prognostic factors (Table IV). However, bearing in mind the different patient populations, the small numbers of patients involved and the overlapping confidence intervals (data not shown), the suggestion of a dose–response relationship for docetaxel remains to be confirmed in prospective, comparative clinical trials.

The median duration of response (34 weeks) and time to progression (24 weeks) achieved with docetaxel in the present study are shorter than the 44+ and 37+ weeks, respectively, reported with the 100 mg m$^{-2}$ dosage in the other European study (Chevallier *et al.*, 1995). A smaller difference was observed for median time to first response (12 *vs* 11 weeks), although a more marked difference has been demonstrated in a pooled analysis of 172 advanced breast cancer patients treated with first-line docetaxel (75 or 100 mg m$^{-2}$) [12 weeks (range 3–35+) *vs* 9 weeks (range 2+–29+)] (data on file [2], Rhône-Poulenc Rorer).

The acute adverse events associated with docetaxel (75 mg $m^{-2}$) in the present study (e.g. nausea and vomiting, diarrhoea, stomatitis) are commonly seen with the majority of the anti-cancer agents and were well tolerated by patients in this study. No toxic deaths occurred.

Despite a high incidence of grade 4 neutropenia (82% of patients, 53% of cycles), febrile neutropenia was observed in only three patients, during three cycles, and none of these required hospitalisation. No grade 3 or 4 infections were observed. The discrepancy between a high incidence of grade 4 neutropenia and a lower incidence of its complications is probably related to the short period of grade 4 neutropenia, which lasted for more than 7 days in only 3.5% of cycles in which it occurred. The severity of neutropenia was only slightly higher in the earlier study of docetaxel (100 mg $m^{-2}$) (Chevallier *et al.*, 1995): grade 4 neutropenia occurred in 91% of 34 patients (69% of 177 treatment cycles). However, in the same study, grade 4 neutropenia lasted for more than 7 days in only 1 of 123 evaluable cycles (data on file [3], Rhône-Poulenc Rorer) and no grade 3 or 4 infection was observed during neutropenia.

Compared with docetaxel (100 mg $m^{-2}$) (Chevallier *et al.*, 1995), fewer hypersensitivity reactions occurred in the present study (29% *vs* 13%), despite the fact that no routine premedication was used.

Fluid retention was the most frequently reported chronic adverse event associated with docetaxel. This syndrome was cumulative, non-life-threatening effect and it was slowly reversible after treatment discontinuation (median time to resolution 18 weeks). No cardiac, renal and endocrinological disorders that might have been responsible for fluid retention were observed. Recent data suggest that the pathophysiological mechanism may be related to an increase in vascular permeability (data on file [4], Rhône-Poulenc Rorer). Fluid retention was a reason for study discontinuation in 16 (41%) patients at a median cumulative dose of 592 mg $m^{-2}$. Nevertheless, 12 of these 16 patients showed a confirmed objective response, indicating that the development of fluid retention does not interfere with the anti-tumour response to docetaxel. Furthermore, fluid retention was not associated with any significant deterioration in performance status in comparison with baseline measurements. The incidence of fluid retention was similar in the present study and the earlier study of docetaxel (100 mg $m^{-2}$) (72% *vs* 77%), and led to treatment discontinuation in a comparable percentage of patients (41% with 75 mg $m^{-2}$ and 50% with 100 mg $m^{-2}$). Likewise, the median cumulative dosage at treatment discontinuation because of fluid retention was approximately 600 mg $m^{-2}$ in both studies. Interestingly, the cumulative dosage of docetaxel at onset of fluid retention was approximately 100 mg $m^{-2}$ lower with the 75 mg $m^{-2}$ dosage than with the 100 mg $m^{-2}$ dosage (227 mg $m^{-2}$ *vs* 322 mg $m^{-2}$), although this may be explained by the fact that the investigators were more aware of the likelihood of diagnosing oedema in the current study.

In conclusion, the results of this study of docetaxel, at a dosage of 75 mg $m^{-2}$ as a 1 h intravenous infusion every 3 weeks, confirm the efficacy of docetaxel as a first-line chemotherapeutic agent for the treatment of patients with metastatic breast cancer. Comparing the response rates in this study with those reported with the usual dose of 100 mg $m^{-2}$ in a similar study of first-line chemotherapy, it appears that 75 mg $m^{-2}$ produces lower response rates than the higher dosage (51.6% *vs* 67.7% respectively). Nevertheless, the anti-tumour activity of docetaxel (75 mg $m^{-2}$) appears similar to that of first-line doxorubicin monotherapy at the recommended dose of 60–75 mg $m^{-2}$ (Henderson, 1991). The trend in the dose–effect response with docetaxel still requires further confirmation in prospective studies.

The incidence and severity of fluid retention were similar in the present study of docetaxel (75 mg $m^{-2}$) and the earlier study of 100 mg $m^{-2}$, but fewer hypersensitivity reactions appeared to occur at the lower dosage. There is now some evidence to suggest that prophylactic corticosteroid-based premedication for hypersensitivity reactions and peripheral oedema may be useful both in reducing the incidence and severity of these events (Schrijvers *et al.*, 1993; Valero *et al.*, 1994) and in delaying the onset of fluid retention. It is possible that this strategy in combination with docetaxel (100 mg $m^{-2}$) will be preferable to a reduced docetaxel dosage schedule for improving the safety profile of this drug while retaining maximum response rates.

**Acknowledgement**
The author would like to thank Rhône-Poulenc Rorer Pharmaceuticals, France, for supplies of docetaxel and study sponsorship.

### References

ANTMANN K AND SOUHAMI RL. (1993). High dose chemotherapy in solid tumors. *Ann. Oncol.*, **4**, S29–S44.

BISSERY MC, GUENARD D, GUÉRITTE VOEGELEIN F AND LAVELLE F. (1991). Experimental antitumour activity of Taxotere® (RP 56976, NSC 628503), a taxol analog. *Cancer Res.*, **51**, 4845–4852.

BISSETT D, SETANOIANS A, CASSIDY J, GRAHAM MA, CHADWICK GA, WILSON P, AUZANNET V, LE BAIL N, KAYE SB AND KERR DJ. (1993). Phase I and pharmacokinetic study of Taxotere® (docetaxel) (RP 56976) administered as a 24 hour infusion. *Cancer Res.*, **53**, 523–527.

BURRIS HA, IRVIN R, KUHN J, KALTER S, SMITH L, SHAFFER D, FIELDS S, WEISS G, ECKARDT J, RODRIGUEZ G, RINALDI D, WALL J, COOK G, SMITH S, VREELAND F, BAYSSAS M, LE BAIL N AND VON HOFF D. (1993). Phase I clinical trial of Taxotere® (docetaxel) given as either a 2-hour or 6-hour infusion. *J. Clin. Oncol.*, **11**, 950–958.

CHEVALLIER B, FUMOLEAU P, KERBRAT P, DIERAS V, ROCHE H, KRAKOWSKI I, AZLI N, BAYSSAS M, LENTZ MA AND VAN GLABBEKE M. (1995). Docetaxel is a major cytotoxic drug for the treatment of advanced breast cancer: a phase II trial of the Clinical Screening Cooperative Group of the European Organization for Research and Treatment of Cancer. *J. Clin. Oncol.*, **13**, 214–222.

CHEBLOWSKI RT, SMALLEY RV, WEINER JM, IRWIN LE, BARTOLUCCI AA AND BATEMAN JR. (1989). Combination versus sequential single agent chemotherapy in advanced breast cancer: associations with metastatic sites and long term survival. *Br. J. Cancer*, **59**, 227–230.

CLARK GM, SLEDGE GW JR, OSBORNE CK AND McGUIRE WL. (1987). Survival from first recurrence: relative importance of prognostic factors in 1015 breast cancer patients. *J. Clin. Oncol.*, **5**, 55–61.

Data on file [1], Rhône-Poulenc Rorer. Docetaxel. Investigators' Brochure, 30 November 1994.

Data on file [2], Rhône-Poulenc Rorer. Riva A. Metastatic breast cancer. Update of North American and European studies, October 1994 (updated Integrated Efficacy Analysis).

Data on file [3], Rhône-Poulenc Rorer. Final Medical Report, study TAX237.

Data on file [4], Rhône-Poulenc Rorer. Bizzari JP, Le Bail N. Integrated Safety Summary – Updated Analysis, October 1994.

Data on file [5], Rhône-Poulenc Rorer. Final Medical Report, study TAX266.

Data on file [6], Rhône-Poulenc Rorer. Final Medical Report, study TAX228.

DE VALERIOLA D, BRASSINNE C, PICCART M, TOMIAK E, KERGER J, AWADA A, RAVOET C, LIPS S, AUZANNET V, LE BAIL N, BRUNO R AND KLASTERSKY J. (1992). Phase I pharmacokinetic study of Taxotere® (RP 56976, NSC 628503) administered as a weekly infusion (abstract). *Proc. Am. Assoc. Cancer Res.*, **33**, 1563.

EXTRA JM, ROUSSEAU F, BRUNO R, CLAVEL M, LE BAIL N AND MARTY M. (1993). Phase I and pharmacokinetic study of Taxotere® (RP 56976; NSC 628503) given as a short intravenous infusion. *Cancer Res.*, **53**, 1037–1042.

FISHER B, SLACK N, KATRYCH D AND WOLMARK N. (1975). Ten year follow-up results of patients with carcinoma of the breast in a co-operative clinical trial evaluating surgical adjuvant chemotherapy. *Surg. Gynecol. Obstet.*, **140**, 528–534.

FLEMING T. (1982). One-sample multiple testing procedure for phase II clinical trials. *Biometrics*, **38**, 143–151.

HANAUSKE AR, DEGEN D, HILSENBECK SG, BISSERY MC AND VON HOFF DD. (1992). Effects of Taxotere® and Taxol on *in vitro* colony formation of freshly explanted human tumor cells. *Anti-Cancer Drugs*, **3**, 121–124.

HARRIS JR, LIPPMAN ME, VERONESI U AND WILLETT W. (1992). Breast cancer (first of three parts). *N. Engl. J. Med.*, **327**, 319–328.

HARRIS JR, MORROW M AND BONADONNA G. (1993). Cancer of the breast. In *Cancer: Principles and Practice of Oncology*, 4th ed. De Vita T Jr, Hellman S, Rosenberg SA. (eds) pp. 1264–1332. JB Lippincott Co: Philadelphia.

HENDERSON IC. (1991). Chemotherapy for metastatic disease of breast cancer. In *Breast Disease*, 2nd ed. Harris J, Hellman S, Henderson IC, Kinne DW. (eds) pp. 604–665. JB Lippincott Company: Philadelphia.

HORTOBAGYI GN AND BUZDAR AU. (1993). Present status of anthracyclines in the adjuvant treatment of breast cancer. *Drugs*, **45** (suppl. 2), 10–19.

INOUE K, OGAWA H, HORIKOSHI N, AIBA K, MUKAIYAMA T, MIZUNUMA N, ITAMI S, HIRANO A, MATSUOKA A AND MATSUMURA T. (1991). Evaluation of prognostic factors for 233 patients with recurrent advanced breast cancer. *Jpn. J. Clin. Oncol.*, **21**, 334–339.

JENSEN OM, ESTEVE J, MOLLER H AND RENARD H. (1990). Cancer in the European Community and its member states. *Eur. J. Cancer*, **26**, 1167–1256.

MCPHERSON K, STEEL CM AND DIXON JM. (1994). Breast cancer – epidemiology, risk factors, and genetics. *Br. Med. J.*, **309**, 1003–1006.

PAZDUR R, NEWMAN RA, NEWMAN BM, FUENTES A, BENVENUTO J, BREADY B, MOORE D, JAIYESIMI I, VREELAND F, BAYSSAS M AND RABER MN. (1992). Phase I trial of Taxotere®: five-day schedule. *J. Natl Cancer Inst.*, **84**, 1781–1788.

RINGEL I AND HORWITZ SB. (1991). Studies with RP 56976 (Taxotere): a semisynthetic analogue of taxol. *J. Natl Cancer Inst.*, **83**, 288–291.

ROSS MB, BUZDAR AU, SMITH TL, ECKLES N, HORTOBAGYI GN, BLUMENSCHEIN GR, FREIREICH EJ AND GEHAN EA. (1985). Improved survival of patients with metastatic breast cancer receiving combination chemotherapy. *Cancer*, **55**, 341–346.

SCHRIJVERS D, WANDERS J, DIRIX L, PROVE A, VONCK I, VAN OOSTEROM A AND KAYE S. (1993). Coping with toxicities of docetaxel (Taxotere™). *Ann. Oncol.*, **4**, 610–611.

SEIDMAN AD, HUDIS C, CROWN JPA, BALMACEDA C, LEBWOHL D, CURRIE V, GILEWSKI T, HAKES T, ROBLES M, KLEM K, LEPORE J AND NORTON L. (1993). Phase II evaluation of Taxotere® (RP 56976; NSC 628503) as initial chemotherapy for metastatic breast cancer (abstract). *Proc. Am. Soc. Clin. Oncol.*, **12**, 52.

SIMON R. (1986). Confidence intervals for reporting results of clinical trials. *Ann. Intern. Med.*, **105**, 429–435.

SIMON R. (1989). Optimal two-stage design for phase II clinical trials. *Controlled Clinical Trials*, **10**, 1–10.

TRUDEAU ME, EISENHAUER E, LOFTERS W, NORRIS B, MULDAL A, LETENDRE F, VANDENGURG T AND VERMA S. (1993). Phase II study of Taxotere® as first line chemotherapy for metastatic breast cancer (MBC). A National Cancer Institute of Canada Clinical Trials Group (NCIC CTG) study (abstract). *Proc. Am. Soc. Clin. Oncol.*, **12**, 59.

VALERO V, WALTERS R, THERIAULT R, ESPARZA L, HOLMES F, FRASCHINI G, PLASSE T, BELLET R, RABER M, BUZDAR A AND HORTOBAGYI G. (1994). Phase II study of docetaxel (Taxotere®) in anthracycline-refractory metastatic breast cancer (abstract). *Proc. Am. Soc. Clin. Oncol.*, **13**, 470.

WILLIAMS SF, MICH R, DESSER R, GOLICK J, BESCHORNER J AND BBITRAN JD. (1989). High-dose consolidation therapy with autologous stem cell rescue in stage IV breast cancer. *J. Clin. Oncol.*, **7**, 1824–1830.